# Fractalkine aggravates LPS‐induced macrophage activation and acute kidney injury via Wnt/β‐catenin signalling pathway

**DOI:** 10.1111/jcmm.16707

**Published:** 2021-06-07

**Authors:** Qiming Gong, Yan Jiang, Xiuhong Pan, Yanwu You

**Affiliations:** ^1^ Department of Nephrology Affiliated Hospital of Youjiang Medical University for Nationalities Baise China; ^2^ Science laboratory Youjiang Medical University for Nationalities Baise China

**Keywords:** acute kidney injury, fractalkine, lipopolysaccharide, macrophage, Wnt/β‐catenin signalling

## Abstract

Fractalkine (CX3CL1, FKN), a CX3C gene sequence inflammatory chemokine, has been found to have pro‐inflammatory and pro‐adhesion effects. Macrophages are immune cells with a critical role in regulating the inflammatory response. The imbalance of M1/M2 macrophage polarization can lead to aggravated inflammation. This study attempts to investigate the mechanisms through which FKN regulates macrophage activation and the acute kidney injury (AKI) involved in inflammatory response induced by lipopolysaccharide (LPS) by using FKN knockout (FKN‐KO) mice and cultured macrophages. It was found that FKN and Wnt/β‐catenin signalling have a positive interaction in macrophages. FKN overexpression inhibited LPS‐induced macrophage apoptosis. However, it enhanced their cell viability and transformed them into the M2 type. The effects of FKN overexpression were accelerated by activation of Wnt/β‐catenin signalling. In the in vivo experiments, FKN deficiency suppressed macrophage activation and reduced AKI induced by LPS. Inhibition of Wnt/β‐catenin signalling and FKN deficiency further mitigated the pathologic process of AKI. In summary, we provide a novel mechanism underlying activation of macrophages in LPS‐induced AKI. Although LPS‐induced murine AKI was unable to completely recapitulate human AKI, the positive interactions between FKN and Wnt/β‐catenin signalling pathway may be a therapeutic target in the treatment of kidney injury.

## 1 INTRODUCTION

An inflammatory reaction is a clinical pathological process which acts as a self‐defence mechanism.^1^ The immune system regulates the antigens or the immune cells, which when uncontrolled, can cause serious organ failure and eventually death.^2^ Macrophages kill pathogens and promote adaptive immunity through antigen presentation.^3^ Macrophages play important roles in the biological progression such as immune homeostasis, cytokine signalling transmission and inflammatory response. Identification of the key mechanisms involved in the progression of inflammatory macrophages is urgent and of high demand in order to improve the clinical outcome.

FKN is a CX3C gene sequence chemokine that functions as immune response and adhesion towards its unique receptor on CX3CR1‐expressing immune cells. FKN occurs in two possible forms, a membrane‐bound and a soluble form, which can be converted into each other in vivo.^4^, ^5^ When inflammation occurs, the membrane‐bound FKN is broken down into a soluble form. FKN mobilizes immune cells to accumulate at the site of action. FKN act as adhesion molecules and chemical primers which regulate the development of inflammatory diseases when tissue injury occurs.^6^ Studies suggested that FKN plays critical roles in the pathological process involved in atherosclerosis,^7^ osteoarthritis ^8^ and muscle injury.^9^ However, the molecular mechanism by which macrophages interfere with FKN in regulating the development of inflammatory diseases is rarely reported. Moreover, the specific mechanism has been unclear.

Wnt/β‐catenin is a highly conservative signalling pathway that activates the transcriptional activity through β‐catenin nuclear translocation,^10^ regulates synaptic transmission, cells growth, proliferation, differentiation, adhesion and genetic stability.^11^, ^12^ All of these factors play an important role in the progression of inflammatory diseases such as acute lung injury,^13^ fibrotic disease ^14^ and systemic lupus erythematosus.^15^ However, the potential mechanisms through which Wnt/β‐catenin interacts with FKN in the LPS‐induced inflammatory system have not been reported. Study of the molecular mechanism may provide a new direction for clarifying the molecular mechanism of FKN which regulates the development of inflammatory diseases.

The study utilized LPS‐induced macrophage and acute kidney injury (AKI) mice. The results suggest that LPS‐induced macrophage activation was accelerated by FKN overexpression. However, FKN deficiency prevented LPS‐induced AKI through inhibiting macrophage activation in which Wnt/β‐catenin signalling contributed an essential role.

## 2 MATERIAL AND METHODS

### 2.1 Cell culture and treatment

J774A.1 cells were obtained from Beina Chuanglian Biology Research Institute (BNCC300973, Beijing, China). Cells were cultured in DMEM (Gibco) with 10% foetal bovine serum (Gibco) at 37°C in a 5% CO_2_ incubator. J774A.1 cells were infected with lentiviral vector particle‐CX3CL1 and Ubi‐MCS‐3FLAG‐SV40‐Cherry‐IRES‐negative control according to manufacturer's protocol (Shanghai GeneChem Co., Ltd.) to achieve FKN overexpression. Stable cell lines were selected by applying puromycin in culture medium. Cells were divided into 12 groups as follows: (1) control group; (2) LPS group: cells were infected with LPS (L2880, Sigma‐Aldrich) 1 μg/ml for 12 hours; (3) Wnt3a (Wnt/β‐catenin signalling pathway activator) group: cells were infected with Wnt3a (ab81484, Abcam) 50 ng/ml for 48 hours; (4) ICG‐001(Wnt/β‐catenin signalling pathway inhibitor) group: cells were infected with ICG‐001 (S2662, Selleckchem) 10 μM/ml for 48 hours; (5) Wnt3a + LPS group: cells were pretreated with Wnt3a (50 ng/ml, 36 hours) and then co‐treated with 1μg/ml LPS for 12 hours; (6) ICG‐001 + LPS group: cells were pretreated with ICG‐001(10 μM/ml, 36 hours) and then co‐treated with 1μg/ml LPS for 12 hours; (7) EX‐FKN group; (8) Ex‐FKN + LPS group: EX‐FKN cells infected with LPS (1 μg/ml, 12 hours); (9) EX‐FKN + Wnt3a group: EX‐FKN cells infected with Wnt3a 50 ng/ml for 48 hours; (10) EX‐FKN + ICG‐001 group: EX‐FKN cells were infected with ICG‐001 10 μM/ml for 48 hours; (11) EX‐FKN + Wnt3a + LPS group: EX‐FKN cells were pretreated with Wnt3a (50 ng/ml, 36 hours) and then co‐treated with 1μg/ml LPS for 12 hours; (12) EX‐FKN + ICG‐001 + LPS group: EX‐FKN cells were pretreated with ICG‐001(10 μM/ml, 36 hours) and then co‐treated with 1μg/ml LPS for 12 hours.

### 2.2 Mice

Two to –three‐month‐old (25 ± 3 g) specific pathogen‐free (SPF) WT C57BL/6 mice (FKN^+/+^) and FKN‐KO C57BL/6 mice (FKN^−/−^) were purchased from Shanghai Genechem Animal Co. Ltd (NO. SYXK 2015‐0008). For FKN‐KO mice, CRISPR/Cas9 technology was used to construct sgRNA sequence (CX3CL1 ‐sgRNA1:CTGGCAGGTTATCACGGGTTGGG; CX3CL1‐sgRNA2:TGGCAGTA ACTCATACGTCCTGG) that targets the FKN gene locus. The surviving embryos after injection with CRISPR/Cas9 mRNA were raised to adulthood, and the founders with FKN Knockout were screened. Mice were housed under 12‐h/12‐h light/dark cycle with free access to food and water (ad libitum) for 1 week before the experiments. All experiments followed the animal experimentation ethics at Youjiang Medical University for Nationalities (NO. SYXK 2017‐0004), and all procedures were performed according to the National Institute of Health Guidelines.

Six‐ to eight weeks of mice were randomly grouped (12 groups containing 36 mice):(1) control group, WT Mice intraperitoneal (IP) injection of 500 μl saline per day; (2) LPS group, WT Mice challenged with LPS (10 mg/kg, 24 hours) by IP injection; (3) Wnt3a group, WT Mice Tail vein injection of recombinant Wnt3a protein (2 μg/kg) for 21 consecutive days; (4) ICG‐001 group, WT Mice IP injection of ICG‐001(5 mg/kg) for 7 consecutive days; (5) Wnt3a + LPS group, WT Mice Tail vein injection of recombinant Wnt3a protein (2 μg/kg) for 21 consecutive days, LPS (10 mg/kg) was IP injected into WT mice on the last day; (6) ICG‐001 + LPS group, WT Mice IP injection of ICG‐001(5 mg/kg) for 7 consecutive day plus LPS (10 mg/kg) was IP injected into WT mice on the last day; (7) FKN‐KO group, FKN‐KO Mice IP injection of 500 μl saline per day; (8) FKN‐KO + LPS group, FKN‐KO mice challenged with LPS (10 mg/kg, 24 h) by IP injection; (9) FKN‐KO + Wnt3a group, FKN‐KO Mice Tail vein injection of recombinant Wnt3a protein (2 μg/kg) for 21 consecutive days; (10) FKN‐KO + ICG‐001 group, FKN‐KO Mice IP injection of ICG‐001(5 mg/kg) for 7 consecutive days; (11) FKN‐KO + Wnt3a + LPS group, FKN‐KO Mice Tail vein injection of recombinant Wnt3a protein (2 μg/kg) for 21 consecutive days plus LPS (10 mg/kg) was IP injected into FKN‐KO mice on the last day; (12) FKN‐KO + ICG‐001 + LPS group, FKN‐KO Mice IP injection of ICG‐001(5 mg/kg) for 7 consecutive days, LPS (10 mg/kg) was IP injected into FKN‐KO mice on the last day.

### 2.3 Renal function measurement

All mice serum was collected for creatinine (Scr) and blood urea nitrogen (BUN) assays used to evaluate the renal function. Scr and BUN levels of mice were examined using the Blood Urea Nitrogen Assay kit (C013‐2‐1, Jiancheng Bioengineering Institute) and Creatinine Assay kit (C011‐2‐1, Jiancheng Bioengineering Institute) according to the manufacturer's instructions. Urine samples were collected during a 24 hours period using metabolic cages (Nalgene, Rochester). Urinary protein was measured using a urine protein assay kit (C035‐2‐1, Jiancheng Bioengineering Institute) according to the manufacturer's instructions.

### 2.4 RNA‐sequencing assay

Total RNA was extracted with TRIzol Reagent (Invitrogen). Agarose gel electrophoresis was used to assess RNA integrity. Total RNA was purified with Qia Quick PCR Kit, and PCR amplification was performed. RNA‐seq libraries for sequencing were constructed by Illumina HiSeq^TM^ 2500. ABI Step OnePlus Real‐Time PCR System (Life Technologies) was used for quantification and pooling. The sequence was performed according to the PE150 mode of HiSeq2500.

### 2.5 Co‐Immunoprecipitation (Co‐IP) Assay

J774A.1 cells were lysed with a pre‐cooled IP lysis buffer for 30 minutes. Cell lysates were pre‐processed with magnetic bead and then incubated with FKN antibody or control IgG at 4°C overnight. The antibody was captured on magnetic bead and inspected using Western blots probed with anti‐Wnt‐4 and anti‐β‐catenin antibodies.

### 2.6 Cells viability assay

Cells viability was analysed using a Cell Counting Kit‐8 kit (M4839, AbMole, Beijing, China). J774A.1 cells were seeded into 96‐well plates at 5 × 10^3^ cells per well. Cells were treated with Wnt3a (25, 50 and 75 ng/ml) and ICG‐001 (5, 10 and 15 μg/ml) for 24, 48 and 72 hours. A total of 10 μl CCK‐8 was added to each well and incubated 1h at 37°C in a 5% CO_2_ incubator after the treatment with Wnt3a and ICG‐001. The TriStar LB 941 multimode microplate reader (Berthold Technologies, Germany) at 450 nm was used in OD assays.

### 2.7 Cell apoptosis assay

The fluorescein isothiocyanate‐Annexin V/propidium iodide (FITC‐Annexin V/PI) apoptosis kit (556 547, BD Biosciences) was used to detected cell apoptosis. Cells were seeded into 6‐well plates and cultured for 48 hours. The cells of each group were collected, rinsed three times with 1 × PBS and then centrifuged for 3 minutes. The cells were then adjusted to 1 × 10^6^ cells/ml and incubated for 15 minutes in a binding buffer containing Annexin V‐FITC and PI. The resulting apoptosis was detected through flow cytometry using a FACS Canto II (BD Biosciences) within 1 hours.

### 2.8 ELISA

Supernatants from all samples were collected. Cyclin D1(SBJ‐M0517, Senbeijia Bioengineering Institute,), TNF‐α (E‐EL‐M0049c, Cusabio Biotech Co., Ltd) and ARG‐1 (CSB‐EL002005MO, Cusabio Biotech Co., Ltd) were detected according to manufacturer's instructions using ELISA Kits. The absorbance was measured using the TriStar LB 941 multimode microplate reader at 450 nm.

### 2.9 Western blotting

All samples which had 40 μg total protein were loaded on sodium dodecyl sulphate polyacrylamide gel electrophoresis (SDS‐PAGE) and then transferred to polyvinylidene fluoride (PVDF) membranes. The membranes were incubated overnight at 4°C with rabbit anti‐FKN (DF12376, 1:500, Affinity), rabbit anti‐iNOS (AF0199, 1:500, Affinity), rabbit anti‐Wnt‐4 (DF9040, 1:500, Affinity), rabbit anti‐β‐catenin (AF6266, 1:500, Affinity), rabbit anti‐c‐Myc (AF0358, 1:500, Affinity), rabbit anti‐CyclinD1 (AF0931, 1:500, Affinity), rabbit anti‐TNF‐α (DF7014, 1:500, Affinity), rabbit anti‐IL‐10 (DF0175, 1:500, Affinity), anti‐ARG‐1 (DF6657, 1:500, Affinity), rabbit monoclonal FKN antibody (ab25091, 1:1000, Abcam), rabbit monoclonal anti‐Wnt‐4(ab262696, 1:1000, Abcam), rabbit monoclonal anti‐β‐catenin (ab6302, 1:1000, Abcam), mouse anti‐β‐actin (AF7018, 1:1000, Affinity), mouse anti‐β‐tubulin (AF7010, 1:10 000, Affinity) and mouse anti‐GAPDH (AF7021, 1:1000, Affinity). The membranes were then incubated with goat anti‐rabbit IgG (S0001, 1:5000, Affinity) and goat anti‐mouse IgG (S0002, 1:5000, Affinity) for 50 minutes at room temperature. Then, they were exposed to an enhanced chemiluminescence substrate (KF003, Affinity) and visualized using Tanon‐5200 (Tanon, Shanghai, China).

### 2.10 HE staining and PAS staining

Kidney tissue samples were fixed in 10% formalin and then embedded in paraffin for histopathology. A total of 3‐4 µm serial sections were stained followed by routine de‐waxing and hydration. These sections were then stained with haematoxylin‐imidine red (HE) or periodic acid‐Schiff (PAS) stains according to standard procedures. These sections were visualized using a light microscope (H550S, NIKON).

### 2.11 Immunofluorescence assay

J774A.1 cells (5 × 10^3^ cells/well) were seeded into glass bottom cell culture dishes (IBIDI, Germany) for 72 hours. J774A.1 cells were then washed three times with 1×PBS and fixed in 4% paraformaldehyde at room temperature for 30 minutes. Cells were permeabilized with 1 ml of 0.1% Triton X‐100 for 20 minutes. Cells were incubated with fluorescently labelled primary antibodies (anti‐FKN, anti‐β‐catenin, anti‐iNOS, anti‐ARG‐1 and anti‐β‐tubulin) followed by either goat anti‐rabbit IgG (H + L) FITC‐conjugated antibody (S0008, 1:200, Affinity) or goat anti‐mouse IgG (H + L) Fluor594‐conjugated antibody (S0005, 1:200, Affinity).. Finally, cells were incubated with DAPI for 10 minutes and imaged using an Olympus Fluoview 3000 Confocal Laser Scanning Microscope (FV3000, Olympus and Tokyo).

Mice kidney sections were prepared using standard procedures. The primary antibodies included anti‐F4/80, anti‐CD11b, anti‐iNOS or anti‐ARG‐1 followed by secondary antibody staining. All sections were visualized under Olympus Fluoview 3000 Confocal Laser Scanning Microscope.

### 2.12 Statistical analysis

All data are expressed in the form of means ± standard deviations. SPSS 23.0 and GraphPad Prism 8.0 were used to analyse statistical data. *P* < .05 was considered as statistically significant. Each experiment was repeated three times.

## 3 RESULTS

### 3.1 FKN positively interacted with Wnt/β‐catenin signalling pathway in macrophage

**FIGURE 1:**
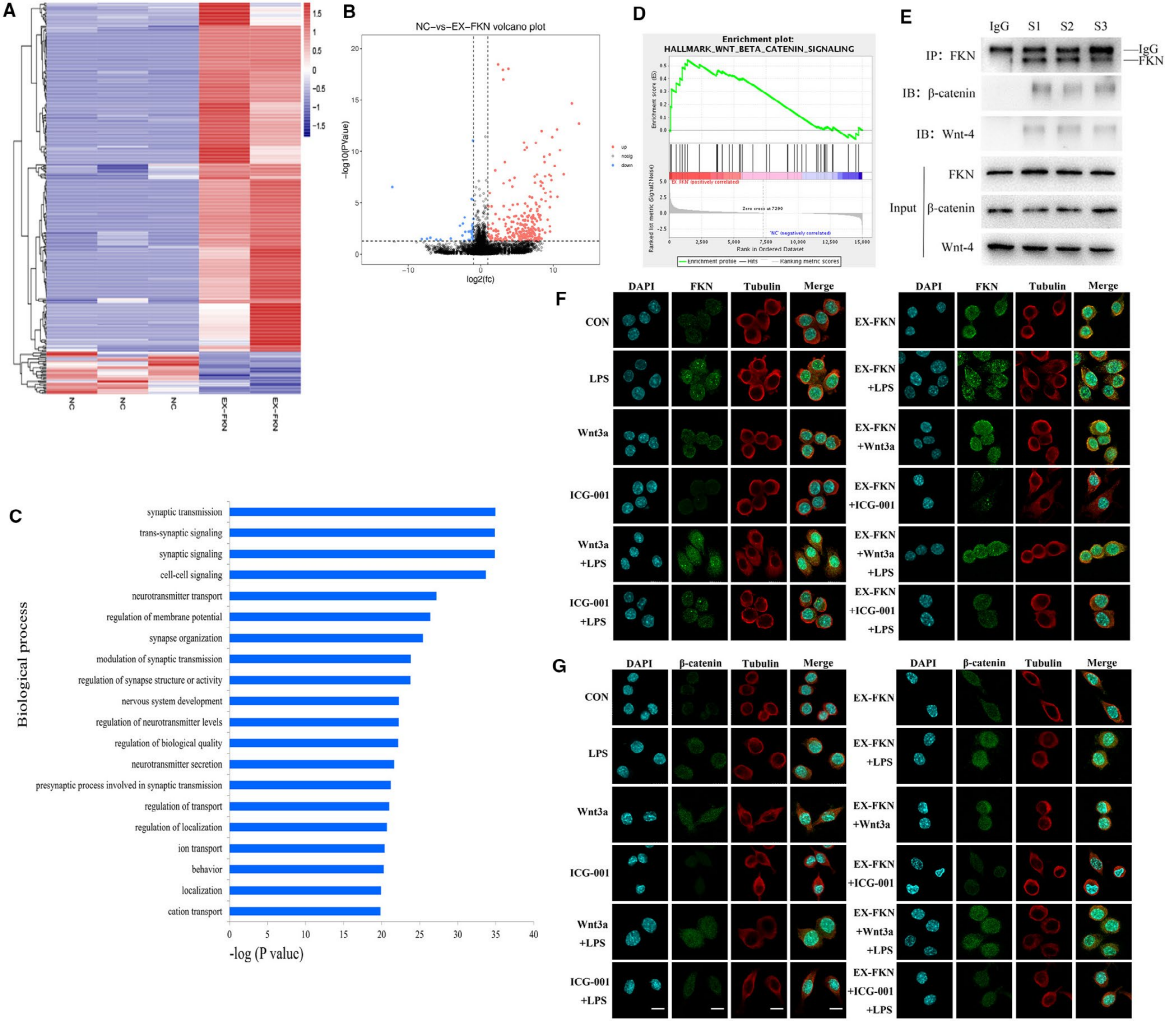
FKN positively interacted with Wnt/β‐catenin signalling pathway in J774A.1 cells. A, Expression profile of FKN‐regulated genes using the RNA‐seq. B, Differentially expressed genes are shown in a volcano map. C, Biological processes were revealed in RNA‐sequencing. D, The data sets were analysed by GESA using the Hallmark collection. The correlation between FKN and Wnt/β‐catenin signalling was indicated in GESA. E‐G, Co‐IP and IF assays detected the interaction between FKN and Wnt/β‐catenin signalling and this was visualized using confocal microscopy with specific antibodies (green). Nuclei were incubated with DAPI (blue). The cytoskeletons were incubated with β‐tubulin (red). Scale bars represent 10 μm

To identify the biological process of FKN in J774A.1 cells, RNA‐sequencing was performed on cells with or without FKN overexpression (Figure 1A,B). The biological process showed that synaptic transmission increased with FKN interference (Figure 1C). GSEA analysis showed that FKN overexpression was positively correlated with the activation of the Wnt/β‐catenin signalling pathway (Figure 1D). J774A.1 cells lysates were collected for co‐IP analysis confirming the interaction between FKN and Wnt/β‐catenin signalling (Figure 1E). FKN and β‐catenin protein distribution were found throughout the cytoplasm and nuclei after IF staining. FKN overexpression enhanced the localization of FKN and β‐catenin protein. Combination treatment with Wnt3a also enhanced the protein localization in J774A.1 cells (Figure 1F,G). A positive interaction between FKN and Wnt/β‐catenin signalling pathway in J774A.1 cells was also confirmed.

### 3.2 FKN promoted LPS‐induced macrophage vitality via Wnt/β‐catenin signalling pathway

**FIGURE 2:**
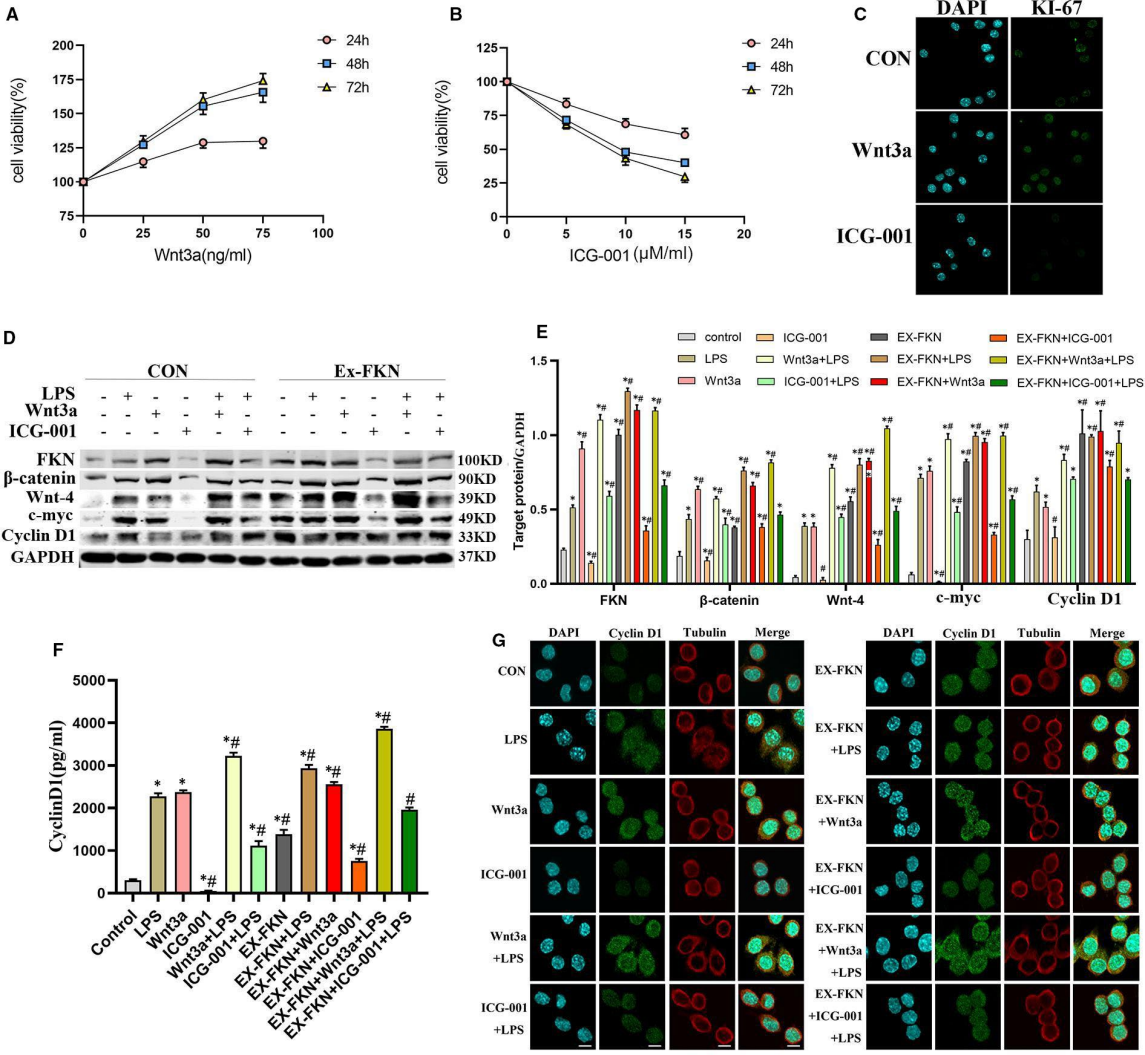
FKN promoted the viability of J774A.1 cells via Wnt/β‐catenin signalling. A, B, Cells were incubated with Wnt3a (25, 50 and 75 ng/ml) and ICG‐001 (5, 10 and 15 μM/ml) for 24, 48 and 72 h. The viability of cells was estimated using the CCK‐8 assay. C, IF assay for KI67 in J774A.1 cells. D, E, Western blotting analysis and their respective quantitation showing the protein expression of FKN, β‐catenin, Wnt‐4, c‐myc and cyclinD1 in J774A.1 cells. F, The secretion of cyclinD1 in J774A.1 cell supernatants was detected using ELISA. ^*^ *P* < .05 compared with the control group; ^#^ *P* < .05 compared with the LPS group. G, The subcellular localization of cyclin D1 was identified by immunostaining using anti‐Cyclin D1 and observed using confocal microscopy. Scale bars represent 10 μm

Cells were treated with different concentrations of Wnt3a (a Wnt/β‐catenin signalling pathway activator) and ICG‐001 (a Wnt/β‐catenin signalling pathway inhibitor) for 48 hours. This helped to assess the role of Wnt/β‐catenin in regulating the vitality of J774A.1 cell. Cells viability was detected using the CCK‐8 assay. Wnt3a increased the viability of macrophages in a concentration‐and time‐dependent manner while ICG‐001 decreased macrophage viability. Wnt3a at 50 ng/ml promoted the proliferation while 10 μM/ml ICG‐001 inhibited proliferation after 48 hours in J774A.1 cells compared to the control group (Figure 2A,B). The expression of Ki67 protein was up‐regulated by Wnt3a but down‐regulated by ICG‐001 in J774A.1 cells after IF staining (Figure 2C). On this basis, Wnt3a at 50 ng/ml and ICG‐001 at 10 μM/ml were used for 48h in the following studies.

C‐myc and cyclin D1 are critical genes involved in cell proliferation and differentiation as downstream targets of classical Wnt/β‐catenin signalling pathway. Results found that LPS increased the expression of FKN, β‐catenin, Wnt‐4, c‐myc and Cyclin D1 protein compared with the controls (*P* < .05). Further research found that c‐Myc and cyclin D1 protein abundance were induced by FKN overexpression. C‐Myc and cyclin D1 combined with Wnt3a to significantly enhance the effects. However, the combination with ICG‐001 reversed the effects in LPS‐induced J774A.1 cells (*P* < .05) (Figure 2D,E). IF staining further showed that LPS increased the amount of cyclin D1 protein in the cytoplasm and nuclei compared with the controls. Wnt3a enhanced the effects of FKN overexpression that increased the cyclin D1 protein localization while this decreased with ICG‐001 (Figure 2G).

### 3.3 FKN inhibited LPS‐induced macrophage apoptosis via Wnt/β‐catenin signalling pathway

**FIGURE 3:**
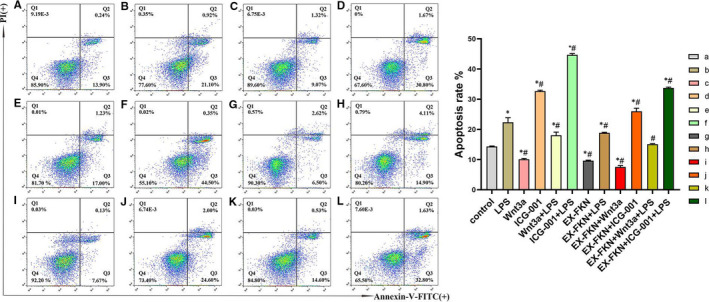
FKN inhibited LPS‐induced apoptosis via Wnt/β‐catenin signalling pathway in J774A.1 cells. ^*^ *P* < .05 compared with the control group; ^#^ *P* < .05 compared with the LPS group

The effects of FKN on the apoptosis of LPS‐induced J774A.1 cells were measured by staining with Annexin V‐FITC/PI for flow cytometry analysis. As shown in Figure 3, the apoptosis rate was increased in the LPS and ICG‐001 groups compared with the control. However, the Wnt3a and EX‐FKN groups inhibited the rate of apoptosis (*P* < .05). Furthermore, the apoptosis rate increased in the ICG‐001 + LPS and EX‐FKN + ICG‐001 + LPS groups compared with LPS group. However, the apoptosis rate was decreased in the Wnt3a + LPS, EX‐FKN + LPS and EX‐FKN + Wnt3a + LPS groups (*P* < .05). The data suggested an anti‐apoptosis of FKN overexpression and Wnt3a in LPS‐induced J774A.1 cells. ICG‐001 played a pro‐apoptosis role in LPS‐induced J774A.1 cells.

### 3.4 FKN transformed LPS‐induced macrophage into M2 phenotype via Wnt/β‐catenin signalling pathway

**FIGURE 4:**
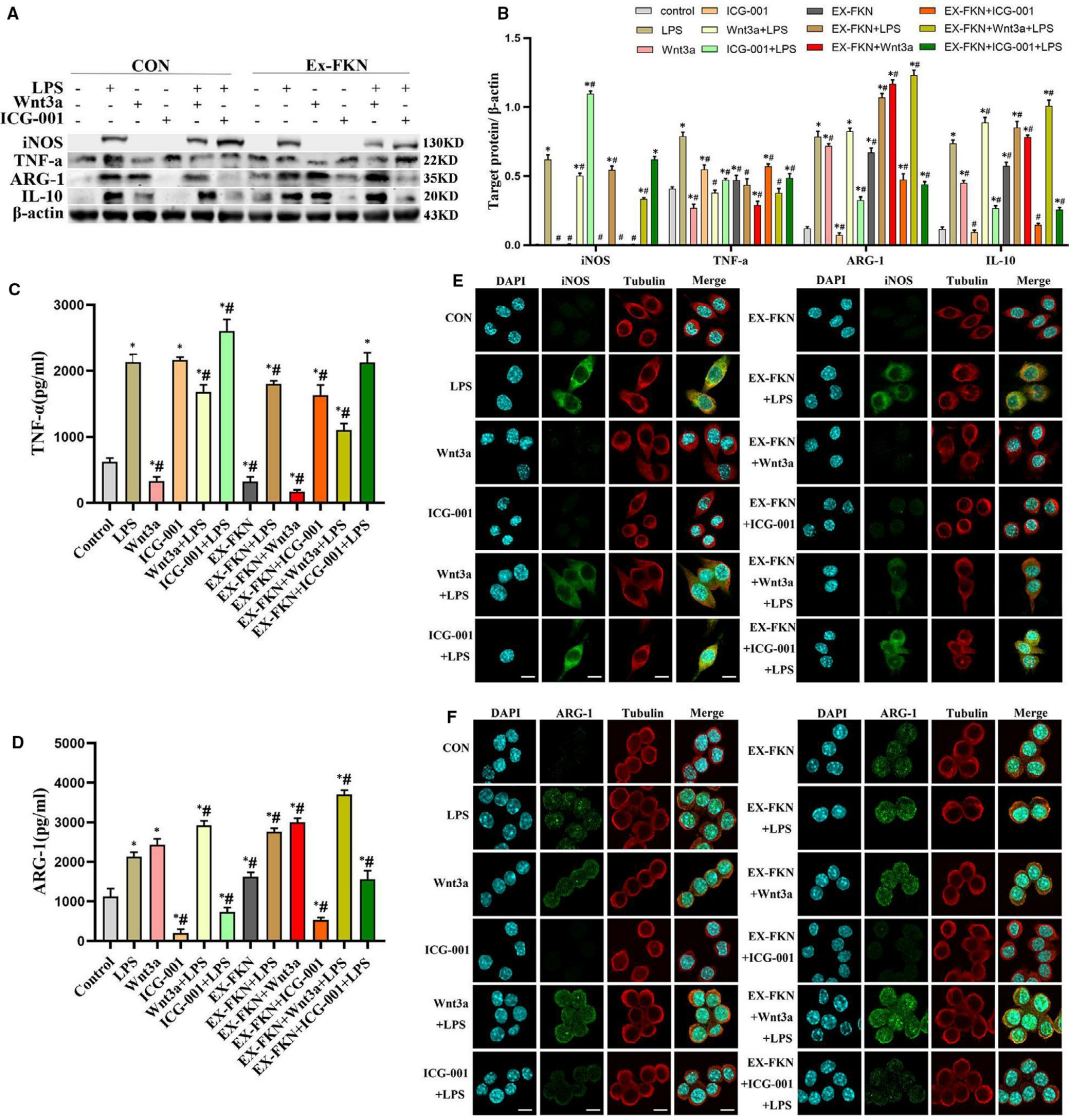
FKN regulated polarization in LPS‐induced J774A.1 cells via Wnt/β‐catenin signalling. A, B, Western blot analysis and their respective quantitation were performed to detect the expression levels of iNOS, TNF‐α, ARG‐1 and IL‐10 protein in J774A.1 cells. C, D, ELISA was used to detect the levels of TNF‐α and ARG‐1 in the cell supernatants. ^*^ *P* < .05 compared with the control group; ^#^ *P* < .05 compared with the LPS group. E, F, Immunofluorescence analysis was used to ascertained the subcellular localization of iNOS and ARG‐1. Scale bars represent 10 μm

Western blotting, ELISA and IF analysis were used to detect the expression of iNOS, TNF‐α, IL‐10, ARG‐1 in each group. This helped to investigate the driving force of FKN on the polarization process of LPS‐induced J774A.1 cells. Figure 4 A‐D showed the low expression of macrophage polarization cytokines iNOS, TNFα, IL‐10 and ARG‐1 in the control group. However, macrophage polarization was highly expressed after LPS treatment (*P* < .05). This confirmed that LPS activated the macrophage polarization process. Furthermore, the Wnt3a and EX‐FKN groups showed a down‐regulation of iNOS and TNF‐α protein. IL‐10 and ARG‐1 protein levels were up‐regulated in LPS‐induced J774A.1 cells (*P* < .05). Combined treatment with Wnt3a (EX‐FKN + Wnt3a + LPS group) markedly strengthened the effects of FKN. However, combined treatment with ICG‐001 (EX‐FKN + ICG‐001 + LPS group) reversed the effects of FKN and further increased the expression of iNOS and TNF‐α in LPS‐induced J774A.1 cells (*P* < .05). Through IF staining, iNOS and ARG‐1 proteins accumulated in both the nuclei and cytoplasm but were mainly located in the cytoplasm after LPS stimulation compared with controls. FKN overexpression and Wnt3a enhanced the localization of ARG‐1 while decreased the localization of iNOS in LPS‐induced J774A.1 cells. ICG‐001 reversed the effects of FKN and Wnt3a (Figure 4 E‐F). The preceding data confirmed that LPS‐induced macrophage towards the M2 phenotype by FKN overexpression and activation of Wnt/β‐catenin signalling pathway.

### 3.5 FKN deficiency attenuated LPS‐induced AKI via inhibition of Wnt/β‐catenin signalling pathway

**FIGURE 5:**
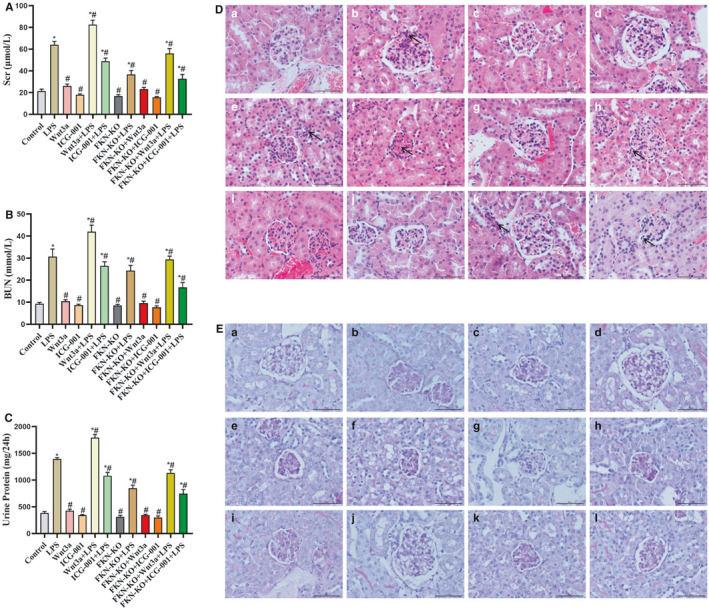
FKN deficiency attenuated LPS‐induced acute kidney injury via inhibition of Wnt/β‐catenin signalling. A‐C, The BUN, serum creatinine and 24 h urinary protein levels. ^*^ *P* < .05 compared with the WT Mice; ^#^ *P* < .05 compared with the LPS Mice. D, E, H&E and PAS stains of kidney tissues. (a) Control mice; (b) LPS mice; (c) Wnt3a mice; (d) ICG‐001 mice; (e) Wnt3a + LPS mice; (f) ICG‐001 + LPS mice; (g) FKN‐KO mice; (h) FKN‐KO + LPS mice;i) FKN‐KO + Wnt3a mice; (j) FKN‐KO + ICG‐001 mice; (k) FKN‐KO + Wnt3a + LPS mice; (l) FKN‐KO + ICG‐001 + LPS mice

The above data from J774A.1 cells demonstrated that overexpression of FKN played essential roles in the pathological progression of LPS‐induced inflammatory response via activation of Wnt/β‐catenin signalling. FKN deficiency attenuated LPS‐induced kidney pathological damage. FKN‐KO mice were selected for the study. Serum creatinine (Scr), blood urine nitrogen (BUN) and 24 hours urinary protein were measured as markers of renal function. As shown in Figure 5A‐C, the levels of Scr, BUN and 24 hours urinary protein in the FKN‐KO + LPS mice were markedly reduced compared with the LPS mice (*P* < .05). ICG‐001‐treated mice (FKN‐KO + ICG‐001 + LPS group) showed further decreased levels. Wnt3a‐treated mice (FKN‐KO + Wnt3a + LPS group) reversed the action of FKN deficiency (*P* < .05). These findings were further supported by the pathological alterations seen in HE‐ and PAS‐stained mice kidney tissues. The renal histopathological findings showed that mice stimulated with LPS exhibited glomerular atrophy, glomerular basement membranes proliferation and inflammatory cells infiltration. However, these renal structural changes in the LPS group were attenuated through intervention with FKN deficiency or treatment with ICG‐001. The combined treatment (FKN‐KO + ICG‐001 + LPS group) further improved the renal damage (Figure 5C,D).

### 3.6 FKN deficiency prevented macrophage proliferation and polarization in LPS‐induced AKI via inhibition of Wnt/β‐catenin signalling pathway

**FIGURE 6:**
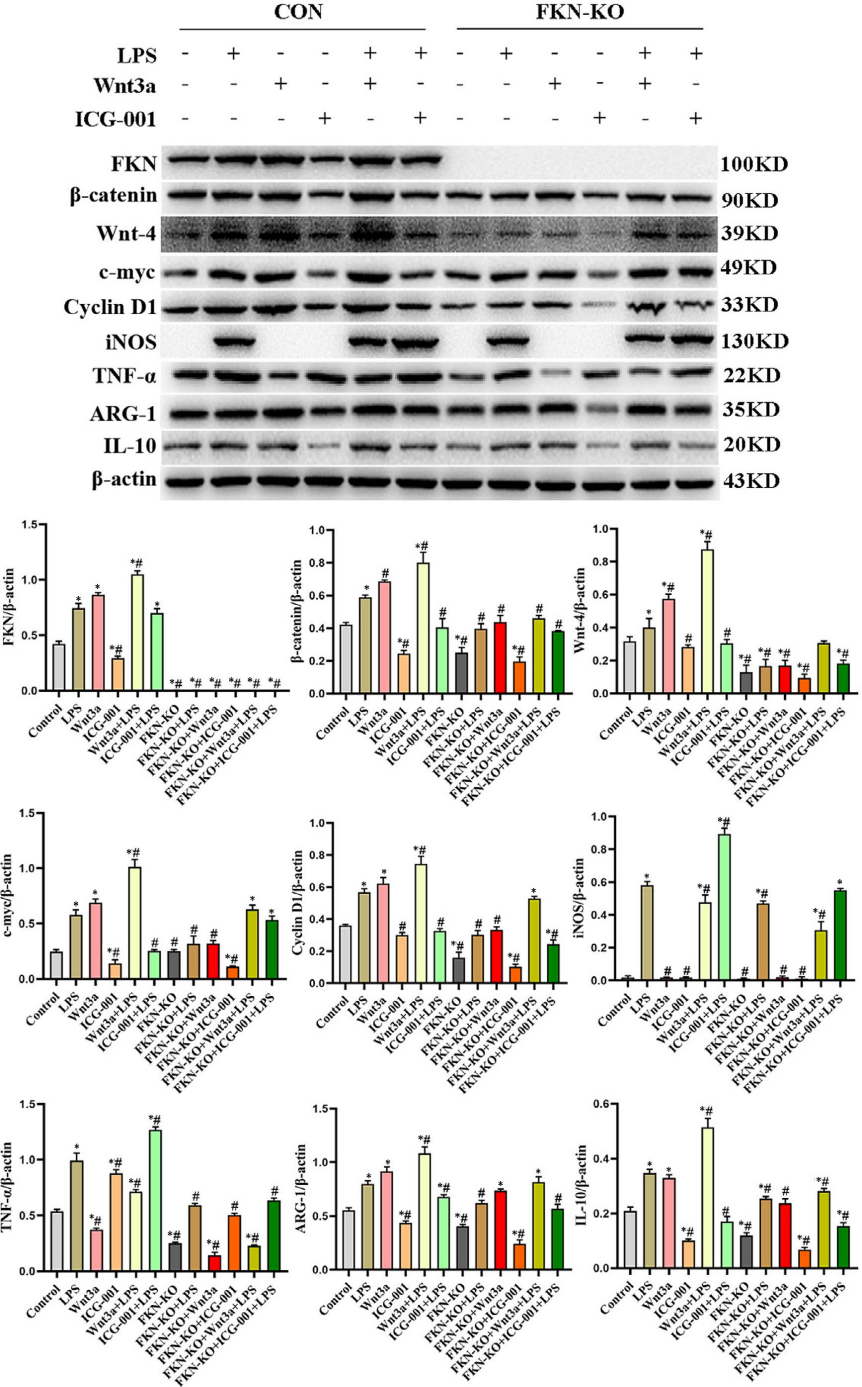
FKN deficiency suppressed macrophages polarization and proliferation in mice kidney tissues via inhibition of Wnt/β‐catenin signalling. A, B, Western blot analysis and their respective quantitation showing the protein expression of FKN, β‐catenin, Wnt‐4, c‐myc, cyclinD1, iNOS, TNF‐α, ARG‐1 and IL‐10 in mice kidney tissues. ^*^ *P* < .05 compared with the WT mice; ^#^ *P* < .05 compared with the LPS mice

Whether FKN deficiency decreased the progression of macrophage proliferation and polarization in the AKI seen after LPS induction was further examined. The protein expression of FKN, β‐catenin, Wnt‐4, c‐myc, cyclin D1, iNOS, TNF‐α, IL‐10 and ARG‐1 was quantified using Western blot analysis. After stimulation with LPS, FKN deficiency (FKN‐KO + LPS group) showed decreased protein expression of FKN, β‐catenin, Wnt‐4, c‐myc, cyclin D1, iNOS, TNF‐α, IL‐10 and ARG‐1 compared with the LPS group (*P* < .05). The combined treatment with ICG‐001 (FKN‐KO + ICG‐001 + LPS group) further inhibited the expression of these proteins. The combined treatment with Wnt3a (FKN‐KO + Wnt3a + LPS group) weakly reversed the effects of FKN deficiency (*P* < .05) (Figure 6).

**FIGURE 7:**
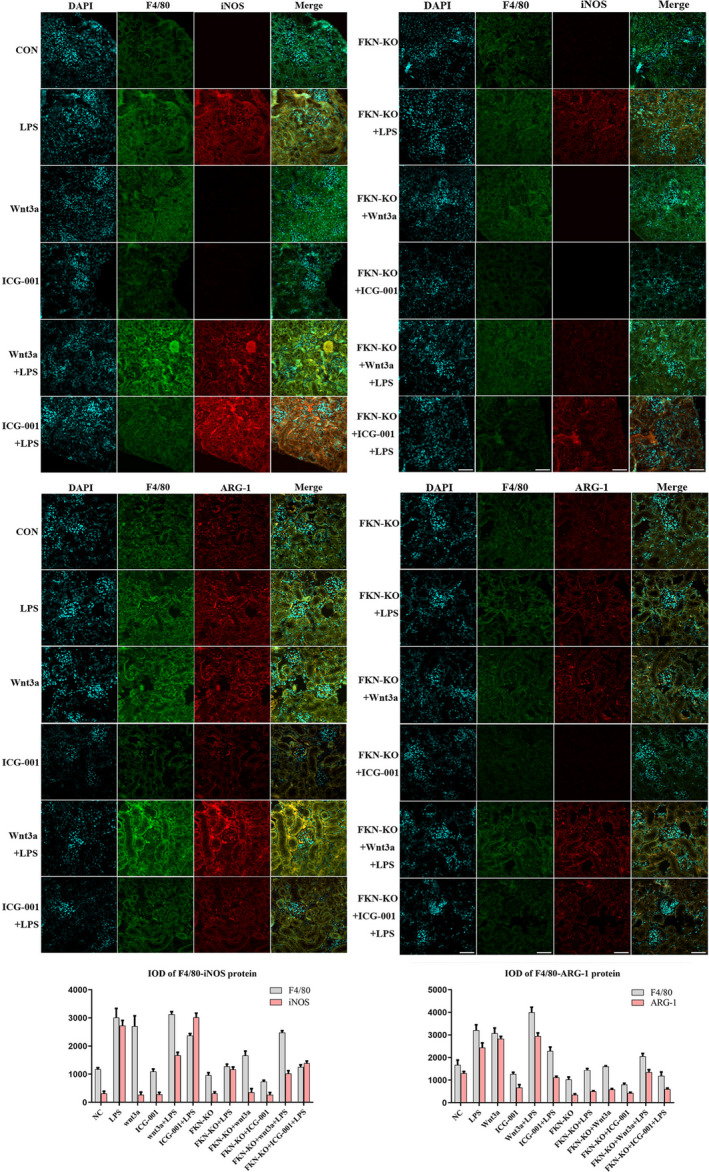
FKN deficiency suppressed F4/80, iNOS and ARG‐1 protein localization in mice kidney tissues via inhibition of Wnt/β‐catenin signalling. Scale bars represent 50 μm

Kidney tissues were stained with antibody F4/80, iNOS or ARG‐1 to identify macrophage accumulation and polarization progression. LPS augmented the F4/80, iNOS or ARG‐1 protein localization in kidney compared with WT mice. Protein localization of F4/80 and ARG‐1 was markedly reduced in the FKN‐KO + LPS group and FKN‐KO + ICG‐001 + LPS group mice kidney tissues compared to LPS group. These results were consistent with Western blotting results. The experimental data showed that FKN deficiency ameliorates LPS‐induced inflammation by suppressing macrophage activation and M2 phenotype differentiation via inhibition of Wnt/β‐catenin signalling (Figure 7).

## 4 DISCUSSION

The mechanism of FKN‐regulated macrophage activation during LPS‐induced inflammatory response is reported in this study. FKN promotes LPS‐induced macrophage activation. The Wnt/β‐catenin signalling pathway plays a major role during macrophage activation process. Furthermore, FKN deficiency suppresses macrophage activation progression by inhibiting Wnt/β‐catenin signalling. This further reduces the pathologic damage of AKI caused by LPS exposure in mice model.

LPS is effective in triggering a robust inflammatory response. LPS is the main microbial mediator leading to septicemia ^16^ which causes tissue injury by accelerating cells necrosis.^17^ LPS is an active component of the cell walls of Gram‐negative bacteria. LPS causes host infection and stimulates macrophage polarization.^18^ Activated macrophages produce several inflammatory cytokines (including iNOS, NO, IL‐6, PGE2, COX‐2 and TNF‐α) which increase the progression of inflammatory response.^19^ Moreover, LPS causes metabolic progression and gradually transforms macrophages into the M2 phenotype. Expression of profibrogenic cytokines (Arg‐1, IL‐10 and TGF‐β) promotes cell differentiation and tissue remodelling ^20^ thereby accelerating the pathological processes of fibrosis disease.^21^ LPS‐induced J774A.1 cells and AKI mice were used as inflammatory model in this study. J774A.1 cells and mice kidney tissue demonstrated a strongly enhanced iNOS, TNF‐α, IL‐10 and ARG‐1 after stimulation with LPS compared with the control group.

The chemokine, FKN, adheres to immune cells during the process of inflammation and preferentially induce the migration of cytotoxic effector lymphocytes which positively correlates with the activity of inflammatory diseases.^22^, ^23^, ^24^ Recent studies have focussed on the therapeutic strategy of FKN in inflammatory diseases. Yu et al found that FKN deficiency ameliorates high fructose diet‐induced kidney injury.^24^ Riopel et al found that FKN expression was elevated in vivo and improved glucose tolerance due to enhanced insulin secretion and decreased β‐cells apoptosis.^25^ In this study, FKN promoted expression of β‐catenin and Wnt‐4 protein (the signature protein of the Wnt/β‐catenin signalling pathway) and inhibited apoptosis in LPS‐induced J774A.1 cells. FKN was highly expressed in the cytoplasm and nuclei in LPS‐induced J774A.1 cells and mice kidney tissue. Furthermore, FKN promotes the secretion of IL‐10 and ARG‐1 but inhibits the secretion of iNOS and TNF‐α in LPS‐induced J774A.1 cells and mice kidney tissue. Mice with FKN deficiency showed decreased expression of c‐myc, Cyclin D1, iNOS, TNF‐α, IL‐10 and ARG‐1. FKN deficiency ameliorates kidney injury through prevention of glomerular atrophy, glomerular basement membranes proliferation and inflammatory cells infiltration.

The molecular mechanism involved in the anti‐inflammatory effects of FKN was further explored based on the study results. The molecular mechanism involved in Wnt/β‐catenin signalling was studied through RNA‐sequencing. This process was altered by FKN. Research showed that abnormal transmission of Wnt/β‐catenin signalling plays an important role in the malignant progression of immune inflammatory diseases by regulating the phagocytosis of macrophages.^26^, ^27^ Activation of Wnt/β‐catenin signalling induced macrophage M2 polarization and promoted the progressive of renal fibrosis.^28^ Inhibition of Wnt/β‐catenin signalling alleviated organ damage caused by sepsis through reducing macrophage infiltration.^29^ However, the study of the relationship between FKN and Wnt/β‐catenin signalling and macrophage activation processes lacked detailed research. In this study, we found a positive interaction between FKN and Wnt/β‐catenin signalling in macrophages. LPS‐induced J774A.1 cells treatment with Wnt3a showed the same capacity with FKN. FKN overexpression combined with Wnt3a enhanced LPS‐induced J774A.1 cell proliferation, anti‐apoptosis and M2 phenotype conversion. For the endotoxin‐shocked wild type mice, ICG‐001 suppressed macrophage proliferation and M2 phenotype conversion which improved the pathological damage of AKI. The anti‐inflammatory role of ICG‐001 was clearly consolidated in the FKN‐KO mice. Study findings suggested that the inhibitory effects of FKN deficiency in inflammation were based on the inhibition of Wnt/β‐catenin signalling pathway.

However, there are some limitations in this paper. Firstly, it is necessary to acknowledge that the LPS‐induced AKI mice model could not mimic all aspects of the human conditions. Secondly, this study focussed on the Wnt/β‐catenin signalling pathway and it is our intention to encompass multiple pathways network in the future. Thirdly, further studies are needed to examine the mechanistic functions of human kidney macrophages in the sepsis inflammatory microenvironment, and ultimately, the therapeutic significance of FKN deficiency should be elucidated in the clinical context.

## 5 CONCLUSIONS

In summary, FKN deficiency exhibits anti‐inflammatory activity in LPS‐stimulated inflammatory mice model through suppression of macrophage proliferation and polarization. Wnt/β‐catenin signalling has a potent biological function through which it exerts its anti‐inflammatory activity. These findings strongly suggest that FKN could be a potential molecular target for the treatment of LPS‐induced inflammatory diseases in the future.

## 6 ETHICS STATEMENT

All animal experiments strictly followed the guidelines of the National Institute of Health and were approved by the Ethics Committee of Youjiang Medical University for Nationalities.

## CONFLICT OF INTEREST

The authors declare that there is no conflict of interest associated with this manuscript.

## AUTHOR CONTRIBUTIONS

**Qiming Gong:** Data curation (equal); Formal analysis (equal); Investigation (equal); Methodology (equal); Resources (equal); Software (equal); Writing‐original draft (equal). **Yan Jiang:** Data curation (equal); Formal analysis (equal); Investigation (equal); Methodology (equal); Resources (equal); Writing‐original draft (equal). **Xiuhong Pan:** Data curation (equal); Formal analysis (equal); Investigation (equal); Methodology (equal); Resources (equal); Writing‐original draft (equal). **Yanwu You:** Conceptualization (lead); Project administration (lead); Writing‐review & editing (lead).

## Data Availability

The RNA sequence data reported in this paper have been deposited in the BioProject database, under accession number PRJNA700110, and these are readily accessible at: http://www.ncbi.nlm.nih.gov/bioproject/700110. The data sets supporting the conclusions of this article are included within the article.
